# Predictive value of the systemic immune-inflammation index combined with the prognostic nutritional index for postoperative recurrence in early-stage cervical cancer: evidence from a multicenter cohort study

**DOI:** 10.3389/fonc.2026.1792373

**Published:** 2026-03-05

**Authors:** Anihenimu Abudoukade, Shihao Hong, Min Jiang, Dong Yin, Burebi Maimaiti

**Affiliations:** 1 Department of Gynecology, Kashigar Regional First People's Hospital, Kashigar, China; 2 Department of Gynecology, Sir Run Shaw Hospital, School of Medicine, Zhejiang University, Hangzhou, China; 3 Department of Oncology, Jining New Journey Cancer Hospital, Jining, China; 4 Department of Oncology, Linyi Hospital of Traditional Chinese Medicine, Linyi, China

**Keywords:** disease-free survival, early-stage cervical cancer, postoperative recurrence, prognostic nutritional index, SII-PNI score, systemic immune - inflammation index, systemic immune-inflammation index

## Abstract

**Background:**

Growing evidence suggests that immune-inflammatory status and nutritional condition influence cancer prognosis. In this study, a composite score combining the systemic immune ~ inflammation index (SII) and the prognostic nutritional index (PNI) was investigated for its ability to stratify the risk of postoperative recurrence in patients with early-stage cervical cancer (ESCC).

**Methods:**

This multicenter, retrospective study enrolled 403 individuals diagnosed with early-stage cervical squamous cell carcinoma and treated with surgery performed with curative intent. Optimal SII and PNI cut-points were selected based on receiver operating characteristic (ROC) curve analysis and validated using internal bootstrapping (1,000 resamples). Disease-free survival was summarized with Kaplan ~ Meier analysis, whereas prognostic associations were quantified using a multivariable Cox proportional hazards model.

**Results:**

ROC-based analyses yielded cutoff values of 580.99 for SII and 49.81 for PNI, which were subsequently used for patient stratification. The combined SII - PNI score showed improved predictive performance compared to individual indices, with an area under the curve (AUC) of 0.743. Survival curves demonstrated a graded increase in postoperative recurrence with rising SII - PNI scores, and intergroup differences reached statistical significance (log-rank P < 0.001). After adjustment for relevant covariates, the combined SII - PNI score remained independently associated with recurrence risk following surgery.

**Conclusion:**

Elevated SII - PNI scores were independently associated with an increased probability of postoperative recurrence among patients with early-stage cervical cancer. Although the discriminatory ability is moderate, this combined index holds promise as a cost-effective, complementary tool for refining risk stratification in this population.

## 1 Introduction

Cervical cancer is one of the most common malignancies among women (1, 2). With the widespread implementation of screening programs and the standardization of diagnostic and therapeutic approaches, detection of early-stage cervical cancer (ESCC) has increased steadily, and surgery remains a primary curative treatment (3). Although most patients with early-stage disease have favorable outcomes, a subset experience postoperative recurrence. Recurrence not only markedly reduces patient survival but also further increases the pressure and burden of subsequent treatment (4, 5). Therefore, a key challenge in current clinical management is how to more accurately identify patients at high risk for recurrence early after surgery, so as to optimize follow-up intensity and adjuvant treatment strategies. Existing recurrence risk assessment relies primarily on pathological and clinical staging variables (tumor size). Although these factors have established prognostic value, their clinical utility is limited by delayed availability and reliance on invasive procedures (6, 7).

Recent studies have increasingly examined the contributions of tumor-related inflammation and patients’ nutritional and immune status to tumor progression and recurrence. Accordingly, peripheral blood ~ based inflammatory and nutritional indices have become a major focus of prognostic research because they are simple, inexpensive, and reproducible (8, 9). Among these, the systemic immune - inflammation index (SII) has been increasingly recognized as a valuable marker for prognostic assessment across a broad range of malignancies (8, 10). Among these, the SII has been increasingly recognized as a valuable marker for prognostic assessment across a broad range of malignancies (11–13). Recent investigations suggest that integrating SII and prognostic nutritional index (PNI) yields additional prognostic information by more effectively capturing the interplay between systemic inflammatory responses and nutritional ~ immune status. In line with this concept, the combined SII - PNI score has been associated with clinical outcomes in severe community-acquired pneumonia, cholangiocarcinoma, gastric carcinoma, and non ~ small-cell lung cancer, highlighting its potential value in practice (14–17). However, data regarding its usefulness for predicting postoperative recurrence in e ESCC are still scarce.

## 2 Methods

### 2.1 Study population

**Figure 1:**
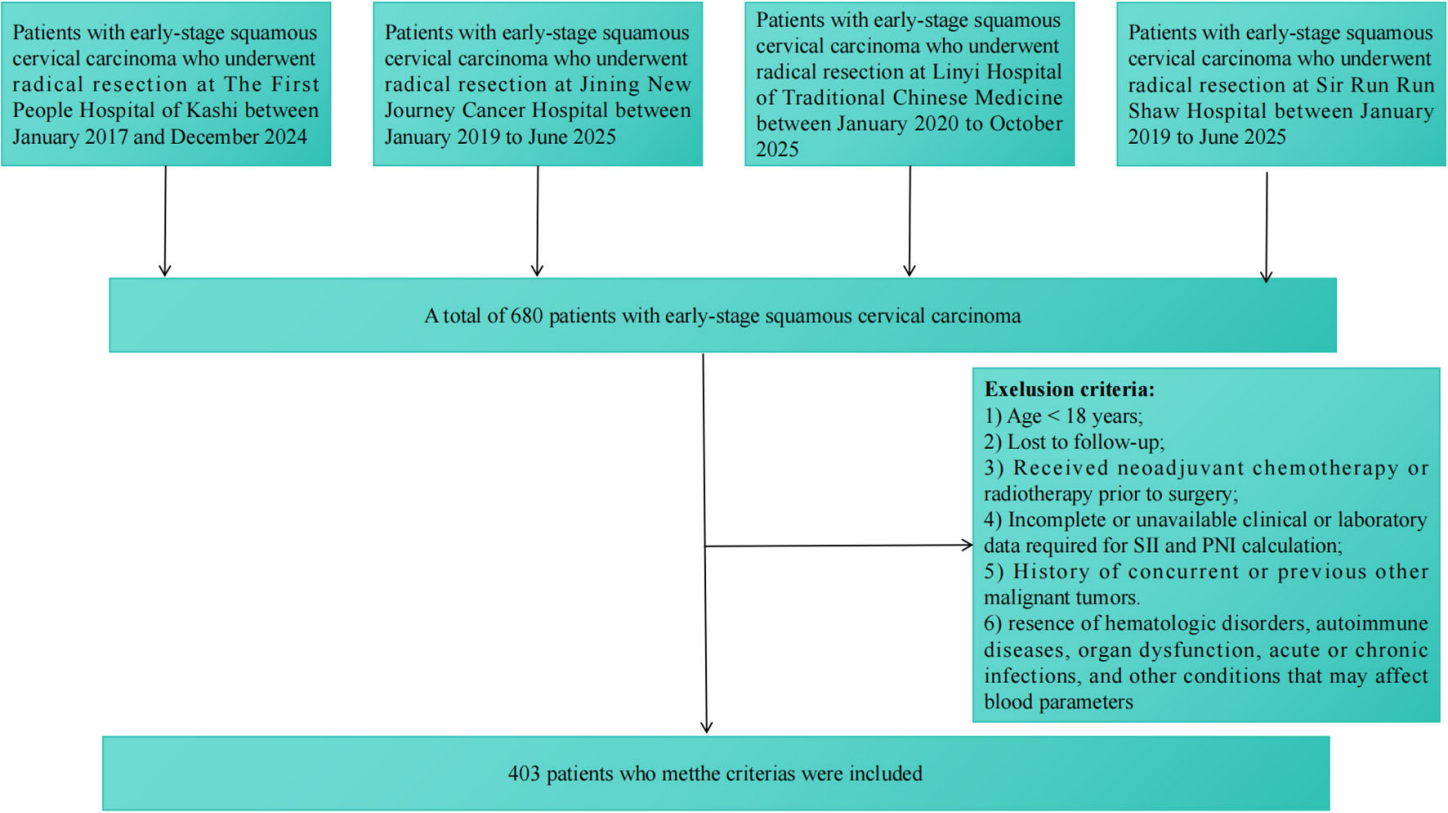
Flowchart of patient inclusion.

This retrospective, multicenter study analyzed patients diagnosed with early-stage cervical squamous cell carcinoma (FIGO stages IA, IB, and IIA1) who underwent radical resection. Data were retrieved from the electronic medical records of four participating institutions. Due to differences in database establishment, the specific data collection periods varied as follows: The First People’s Hospital of Kashi (Jan 2017 - Dec 2024), Jining New Journey Cancer Hospital (Jan 2019 - Jun 2025), Sir Run Run Shaw Hospital (Jan 2019 - Jun 2025), and Linyi Hospital of Traditional Chinese Medicine (Jan 2020 - Oct 2025). To minimize selection bias and ensure representativeness within each center, consecutive enrollment was strictly implemented during each center’s specified data collection window. All patients undergoing radical resection for early-stage cervical squamous cell carcinoma within these defined periods were initially screened for eligibility. Notably, patients with incomplete or unavailable clinical/laboratory data necessary for SII and PNI calculation were excluded, concomitant with other malignant tumors, hematological disorders, autoimmune diseases, or active infections that could affect blood parameters. All diagnoses were histopathologically confirmed as squamous cell carcinoma, and tumor staging was updated according to the 2018 FIGO system. Following this screening process, a total of 403 eligible patients were ultimately included in the final analysis, as detailed in the flowchart (**Figure 1**).

### 2.2 Covariant collection

Clinical data were retrospectively retrieved from the electronic medical record system. Demographic variables included age and body mass index (BMI). Laboratory parameters, including platelet count (PLT), neutrophil count (NEU), lymphocyte count (LYM), and serum albumin levels, were collected from peripheral blood samples obtained within one week prior to surgery to ensure they reflected the preoperative baseline status. Tumor-related characteristics included FIGO 2018 stage (IA, IB, and IIA1), lymphovascular space invasion (LVSI), tumor size and lymph node metastasis status. Information regarding treatment modalities, including radiotherapy and chemotherapy, was also recorded. To ensure consistency with the established PNI formula, which utilizes albumin in g/L, we performed the necessary unit conversion. All albumin values initially reported in g/dL within our dataset were accurately converted to g/L by multiplying by 10 (i.e., Albumin (g/L) = Albumin (g/dL) × 10). The SII and PNI were calculated using the following formulas: SII = (NEU (10^9^/L) × PLT(10^9^/L))/LYM (10^9^/L); PNI = Albumin (g/dl) + 5 × L(10^9^/L). Disease-free survival (DFS) was defined as the time interval from the date of radical surgery to the first documented recurrence. For patients without evidence of recurrence, DFS was censored at the date of the last follow-up.

### 2.3 Statistical analyses

Data distribution was assessed with the Shapiro ~ Wilk test. Variables meeting normality were summarized as mean (SD) and compared between groups using independent-samples t tests. Non-normally distributed variables were presented as median (IQR) and analyzed with the Kruskal-Wallis test. Categorical variables were reported as frequencies and percentages and group differences were evaluated using the χ² test. To assess the prognostic performance for disease-free survival (DFS), a time-to-event outcome with censored data, we employed time-dependent receiver operating characteristic (ROC) curve analysis. Using the time ROC package in R, we calculated the time-dependent area under the ROC curve (AUC) and its 95% confidence interval (CI) at clinically relevant time points, and subsequently determined the optimal cutoff values for SII and PNI based on these analyses. DFS was estimated by the Kaplan ~ Meier method, and differences between groups were evaluated using the log−rank test. Associations between covariates and DFS were examined using univariable and multivariable Cox proportional hazards models, with results reported as hazard ratios (HRs) and 95% confidence intervals (CIs).

To comprehensively evaluate the association of SII and PNI with DFS and explore their potential non-linear relationship, we incorporated SII and PNI into the Cox model as both continuous variables and dichotomous variables determined by the optimal cutoff values from time-dependent ROC curves, respectively. Furthermore, Restricted Cubic Spline (RCS) curves were employed to investigate their non-linear associations. To assess the potential overfitting and validate the stability of the predictive models, internal validation was performed using the bootstrap method with 1,000 resamples. The bias-corrected area under the curve (AUC) was calculated to represent the validated predictive accuracy. All statistical analyses were carried out using R software (version 4.5.1). A two−sided P value < 0.05 was considered statistically significant.

## 3 Results

### 3.1 Baseline demographic and clinical characteristics

In this study, 403 patients were analyzed, the baseline clinicopathological characteristics are summarized in **Table 1**. The mean age at diagnosis was 44 years, with a median follow-up of 31.56 months. Baseline pathological characteristics are summarized in **Table 1**. Lymph node metastasis was observed in 75 patients (18.61%), tumor size greater than 2 cm was recorded in 138 patients (34.24%), and lymphovascular space invasion (LVSI) was identified in 77 patients (19.11%).

**Table 1:** The baseline characteristics of 403 patients with ESCC.

Variable	Total
N	403
Age (years)	44.00 (40.00, 50.00)
BMI (kg/m²)	26.26 (24.01, 29.02)
DFS (month)	28.19 (14.22, 58.29)
PLT (×10^9^/L)	247.00 (202.00, 286.00)
NEU (×10^9^/L)	3.74 (3.07, 4.87)
LYM (×10^9^/L)	1.76 (1.40, 2.10)
Albumin (g/L)	42.11 (39.50, 44.62)
SII	529.04 (358.69, 777.63)
PNI	50.98 (47.75, 54.19)
FIGO 2018, n(%)	
IA	119 (29.53)
IB	127 (31.51)
IIA1	157 (38.96)
Death	
No	380 (94.29)
Yes	23 (5.71)
Chemotherapy, n(%)	
No	335 (83.13)
Yes	68 (16.87)
Radiotherapy, n(%)	
No	303 (75.19)
Yes	100 (24.81)
Tumor size (cm)	
≤ 2	265 (65.76)
> 2	138 (34.24)
LVSI	
No	326 (80.89)
Yes	77 (19.11)
Lymph node metastases	
No	328 (81.39)
Yes	75 (18.61)

Data following a normal distribution are shown as the mean ± SD, while those not normally distributed are depicted as median (IQR) and categorical variables are displayed as count (%%).

BMI, Body mass index; SII, systemic immune- inflammation index; PNI, prognostic nutritional index; PLT, platelet count; NEU, neutrophil count; LYM, lymphocyte count.

### 3.2 ROC curves for PNI and SII

**Figure 2:**
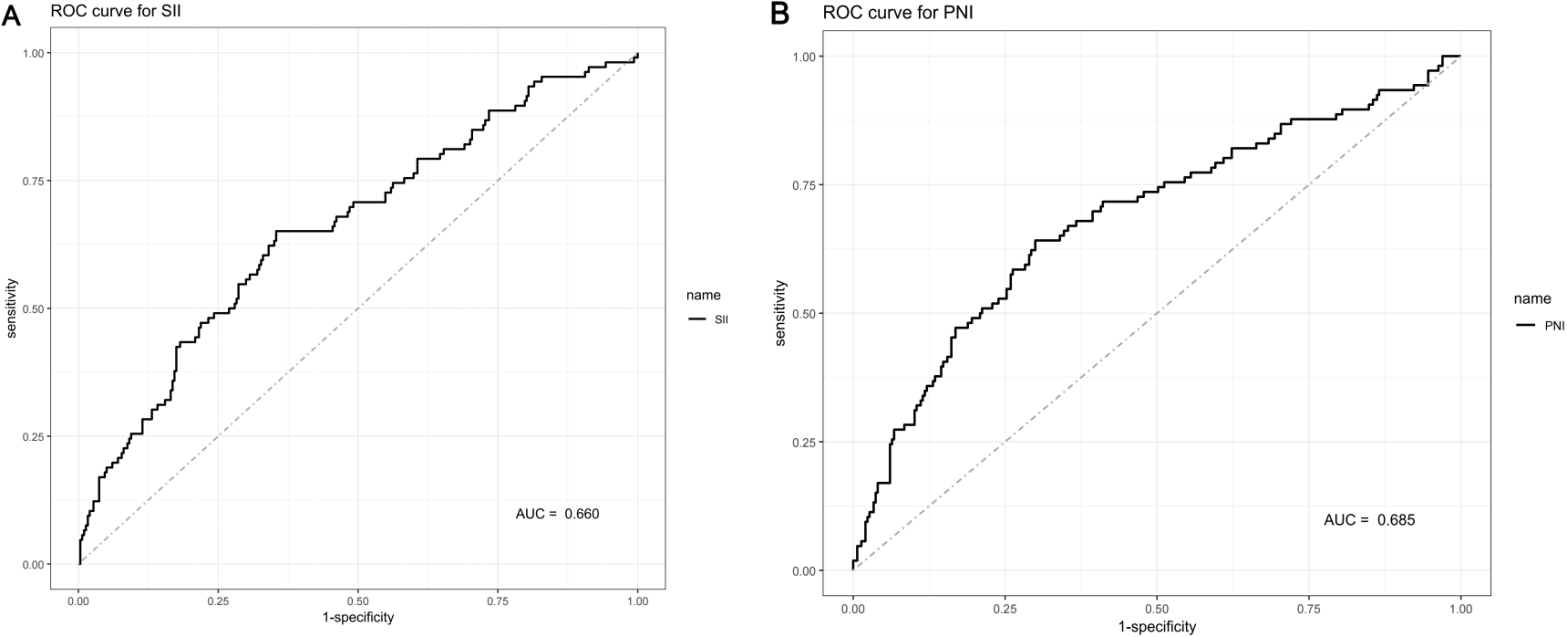
Receiver operating characteristic (ROC) curves for DFS: **(A)** SII; **(B)** PNI.

The discriminatory performance of SII and PNI for predicting postoperative tumor recurrence was assessed via ROC curve analysis (**Figure 2**). The area under the curve (AUC) was 0.660 for SII and 0.685 for PNI. According to the Youden index, the optimal cutoff values were established at 580.99 for SII (Sensitivity: 64.2%; Specificity: 70.0%) and 49.81 for PNI (Sensitivity: 65.1%; Specificity: 64.6%). To assess the robustness of these findings, internal validation was conducted using 1,000 bootstrap resamples. The bias-corrected AUCs were 0.628 for SII (apparent AUC: 0.629) and 0.659 for PNI (apparent AUC: 0.661). The minimal difference between the apparent and bias-corrected values (< 0.005) indicates that the derived cutoff values are stable with negligible overfitting.

### 3.3 Clinical and pathological characteristics stratified by SII - PNI score levels

Based on the established cutoff thresholds, patients were stratified into three groups using a combined SII-PNI scoring system: Score 0: Low SII (≤ 580.99) and High PNI (> 49.81) (n = 139); Score 1: Either High SII (> 580.99) or Low PNI (≤ 49.81) (n = 183); Score 2: High SII (> 580.99) and Low PNI (≤ 49.81) (n = 81). The clinicopathological features of these three groups were compared (**Table 2**). Significant intergroup differences were observed in hematologic indices, including PLT, NEU, and LYM, as well as serum albumin, SII, and PNI levels (P < 0.001). Furthermore, the groups differed significantly regarding key pathological features, including FIGO stage (2018), tumor diameter, LVSI, and lymph node metastasis (P < 0.05). No statistically significant differences were found for other baseline variables.

**Table 2:** The clinicopathological characteristics according to SII-PNI scores.

Variable	SII-PNI=0	SII-PNI = 1	SII-PNI =2	*P*
N	139	183	81	
Age (years)	43.00 (38.00,48.00)	46.00 (40.00,50.00)	45.00 (39.50,50.50)	0.061
BMI (kg/m²)	26.03 (23.65,28.41)	26.67 (24.17,29.30)	26.18 (24.33,30.47)	0.354
DFS (month)	28.33 (12.64,60.31)	25.82 (13.34,59.75)	29.32 (23.54,43.80)	0.353
time (month)	33.06 (12.92,63.30)	30.92 (13.67,66.75)	34.19 (27.05,66.86)	0.091
PLT (×10^9^/L)	229.00 (191.00,258.00)	254.00 (201.00,290.00)	278.00 (244.00,350.00)	<0.001
NEU (×10^9^/L)	3.20 (2.73,3.75)	3.98 (3.20,5.01)	4.96 (3.83,6.53)	<0.001
LYM (×10^9^/L)	1.98 (1.68,2.42)	1.68 (1.32,2.05)	1.58 (1.30,1.81)	<0.001
Albumin (g/L)	43.95 (41.86,46.00)	41.90 (39.58,44.10)	38.70 (36.36,40.26)	<0.001
SII	380.87 (281.74,478.23)	607.55 (380.70,873.17)	856.04 (688.74,1204.09)	<0.001
PNI	53.65 (51.31,56.45)	50.05 (47.32,53.77)	46.72 (44.98,48.46)	<0.001
FIGO 2018, n(%)				0.025
IA	55 (38.46)	50 (27.03)	14 (18.67)	
IB	43 (30.07)	57 (30.81)	27 (36.00)	
IIA1	45 (31.47)	78 (42.16)	34 (45.33)	
Death				0.151
No	134 (96.40)	173 (94.54)	73 (90.12)	
Yes	5 (3.60)	10 (5.46)	8 (9.88)	
Radiotherapy, n(%)				0.014
No	115 (82.73)	135 (73.77)	53 (65.43)	
Yes	24 (17.27)	48 (26.23)	28 (34.57)	
Chemoradiotherapy				0.952
No	116 (83.45)	151 (82.51)	68 (83.95)	
Yes	23 (16.55)	32 (17.49)	13 (16.05)	
Tumor size (cm)				<0.001
≤ 2	111 (77.62)	117 (63.24)	37 (49.33)	
> 2	32 (22.38)	68 (36.76)	38 (50.67)	
LVSI				<0.001
No	126 (88.11)	150 (81.08)	50 (66.67)	
Yes	17 (11.89)	35 (18.92)	25 (33.33)	
Lymph node metastases				0.001
No	122 (85.31)	156 (84.32)	50 (66.67)	
Yes	21 (14.69)	29 (15.68)	25 (33.33)	

BMI, Body mass index; SII, systemic immune- inflammation index; PNI, prognostic nutritional index; PLT, platelet count; NEU, neutrophil count; LYM, lymphocyte count.

The P-value indicates the overall difference among the three groups. Continuous variables were analyzed using the Kruskal-Wallis test, while categorical variables were analyzed using the χ² test.

### 3.4 Predictive value of SII and PNI

**Figure 3:**
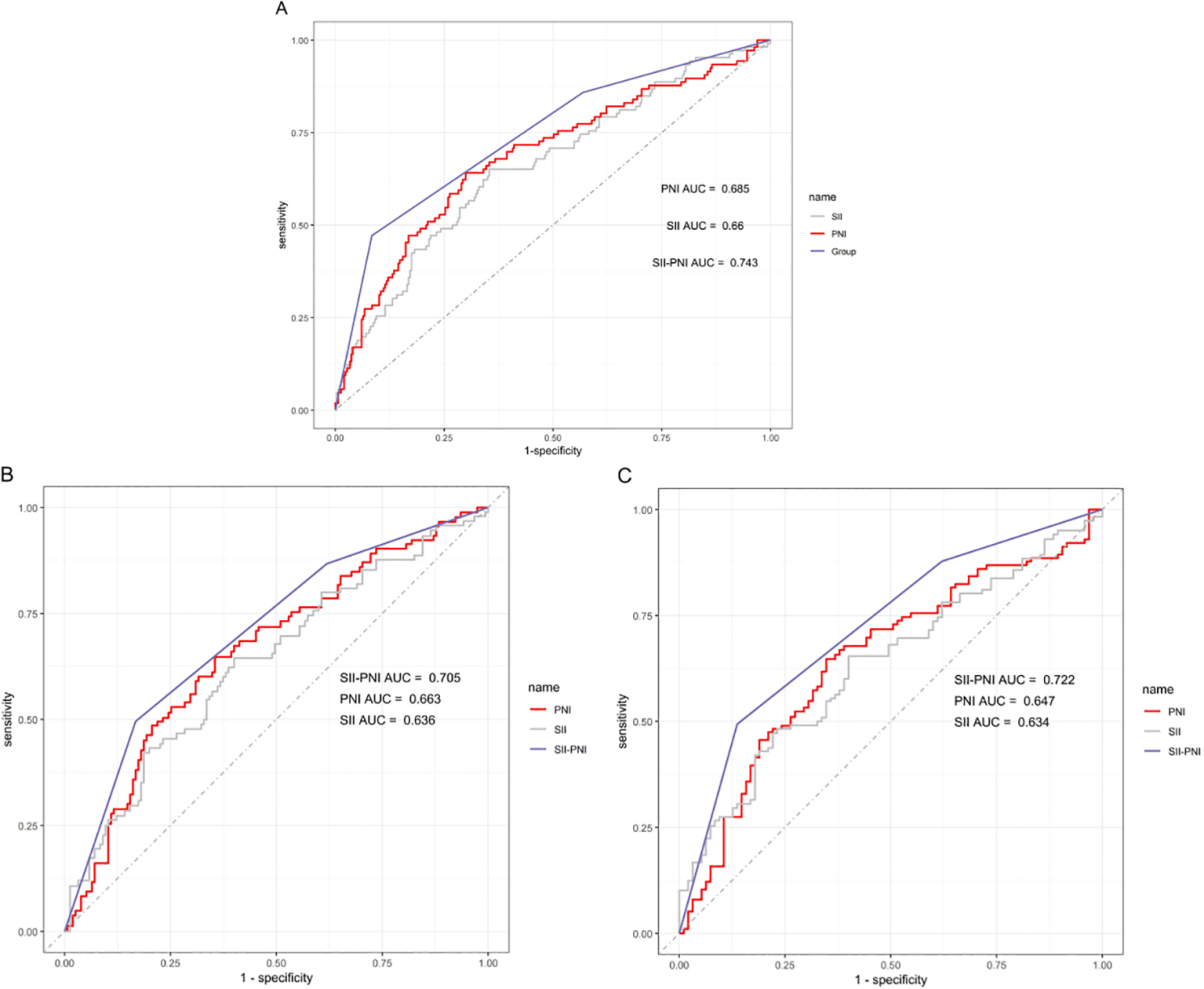
Evaluation of inflammatory indexes as predictors using ROC curves in DFS: **(A)** DFS; **(B)** 3-yeas DFS; **(C)** 5-yeas DFS.

**Figure 4:**
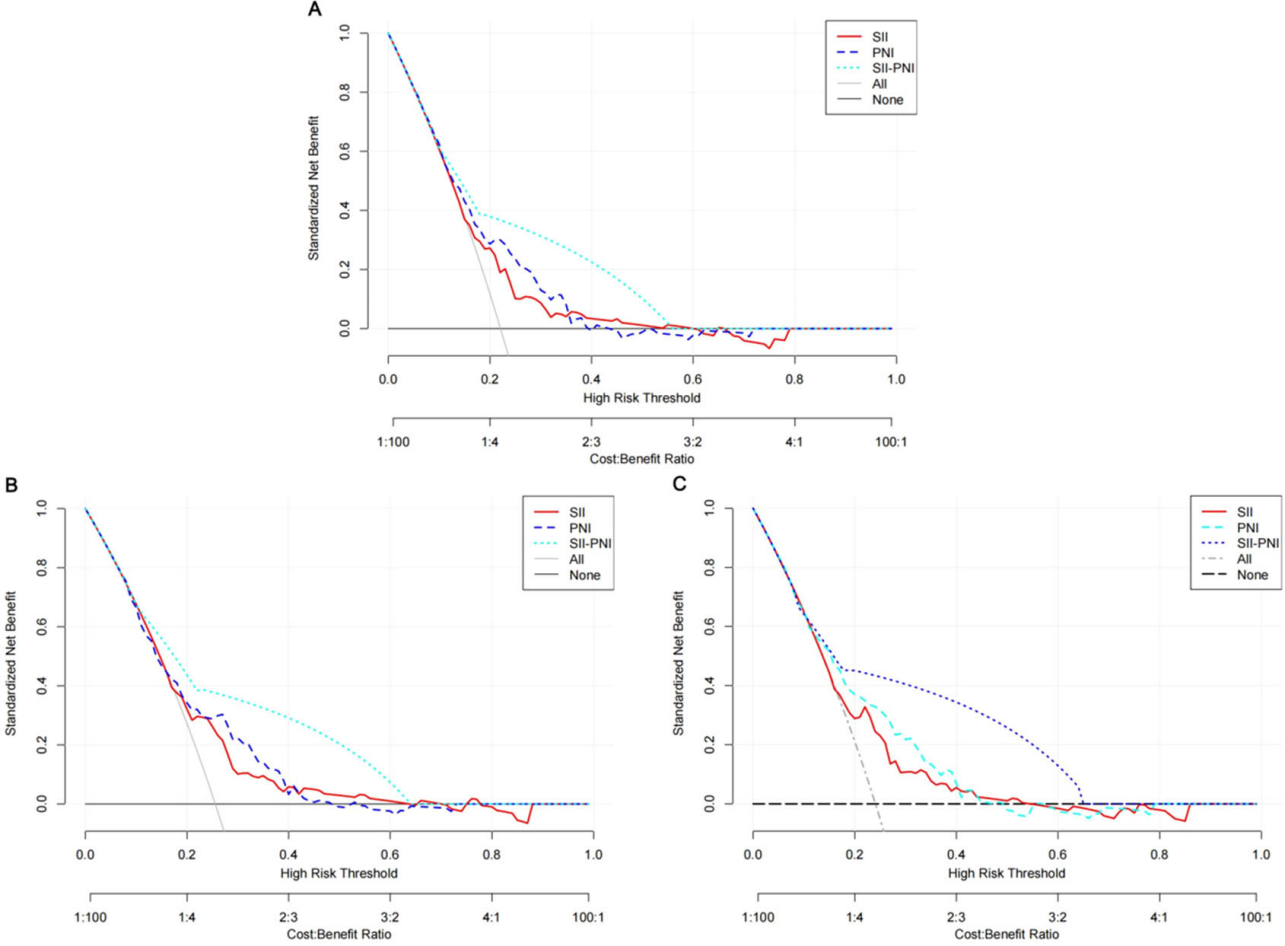
Decision curve analysis for DFS in ESCC patients: **(A)** SII, **(B)** PNI, **(C)** SII-PNI.

When included as continuous variables in a multivariable Cox proportional hazards model, both SII and PNI demonstrated independent prognostic value across different levels of adjustment (**Supplementary Table 1**). After adjusting for various confounding factors, a one standard deviation (SD) increase in SII was significantly associated with an elevated risk of recurrence (HR = 1.17, 95% CI: 1.02 ~ 1.33, P = 0.026). Conversely, a one SD increase in PNI was significantly associated with a reduced risk of DFS (HR = 0.78, 95% CI: 0.64 ~ 0.96, P = 0.016). Furthermore, **Supplementary Figure 1** presents the Restricted Cubic Spline (RCS) analysis results for continuous SII and PNI in relation to recurrence. The RCS analysis revealed a significant non-linear association between both SII and PNI and recurrence. The predictive value of the combined SII-PNI score for recurrence risk was evaluated using ROC curve analysis. The combined SII-PNI score demonstrated superior discriminatory ability compared to SII or PNI alone, achieving an AUC of 0.743 (**Figure 3**). Time-dependent ROC analysis for DFS yielded AUC values of 0.705 at 3 years and 0.722 at 5 years, indicating consistent predictive accuracy across follow-up intervals (**Figure 3**). Additionally, decision curve analysis (DCA) revealed that the combined SII-PNI score provided a higher net benefit than either index alone across a wide range of threshold probabilities, supporting its clinical utility (**Figure 4**).

### 3.5 The impact of combined SII-PNI on DFS

**Figure 5:**
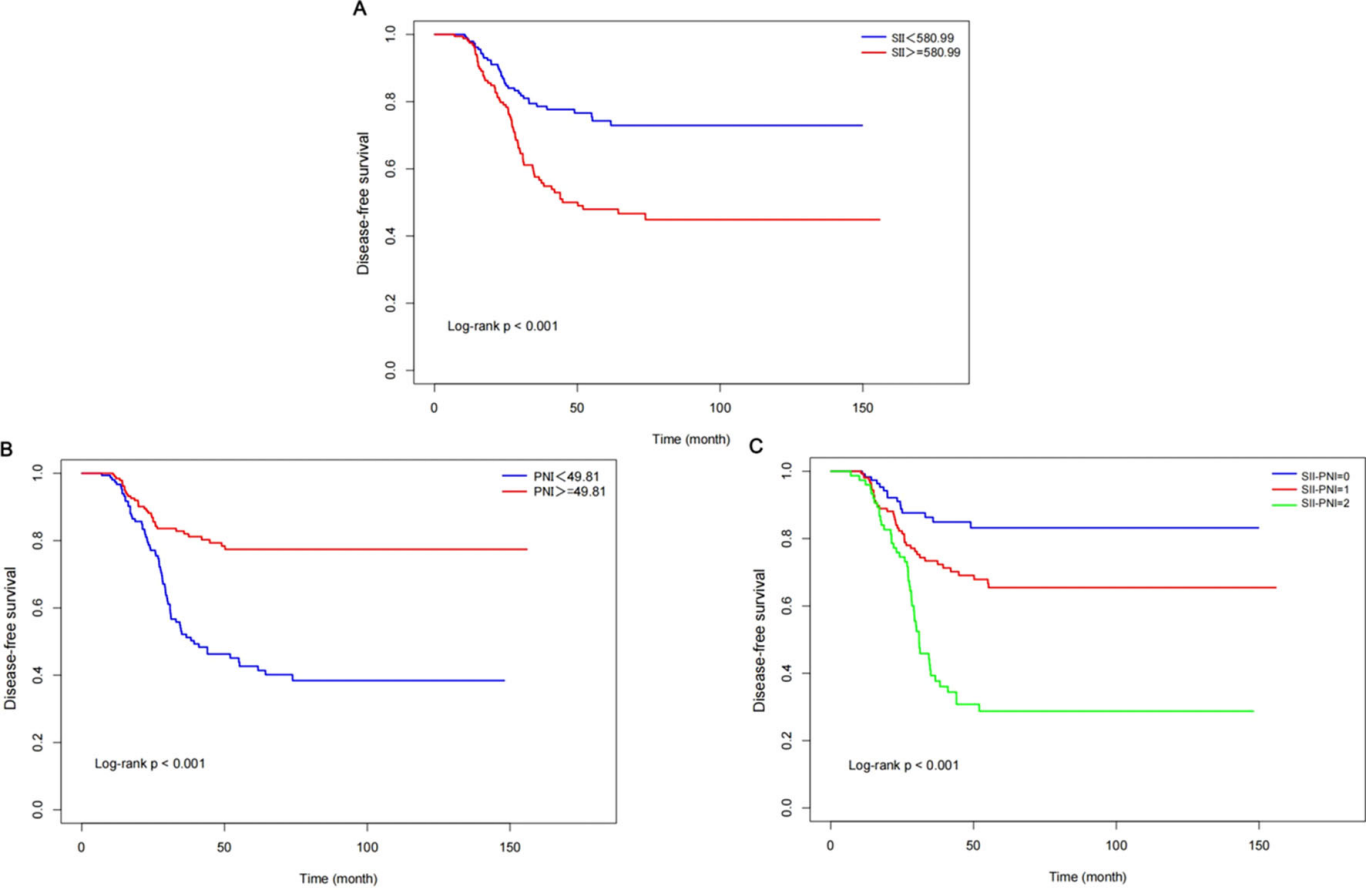
Kaplan-Meier curves for DFS in ESCC patients: **(A)** SII, **(B)** PNI, **(C)** SII-PNI.

Cox regression analysis suggested that score 2 had a higher risk of recurrence compared to score 0 (**Supplementary Table 1**). Kaplan-Meier survival curves stratified by SII levels are shown in **Figure 5**. Patients with high SII exhibited a significantly shorter time to DFS compared with those in the low SII group, indicating an unfavorable prognosis. This difference in DFS was confirmed by log−rank analysis (P < 0.001). consistently, patients with a low PNI exhibited a poorer prognosis and shorter DFS (log-rank test P < 0.001). When SII and PNI were integrated, the SII-PNI score enabled effective risk stratification: patients with a score of 0 showed the most favorable outcomes, those with a score of 1 demonstrated intermediate outcomes, and those with a score of 2 had the poorest outcomes, in line with a progressively increasing risk of recurrence as the combined score rose (log-rank < 0.001).

### 3.6 Univariate and multivariate analyses

Cox proportional hazards regression models were employed for univariate and multivariate analyses of DFS (**Table 3**). Univariate analysis revealed that FIGO stage, the combined SII-PNI score, lymphovascular space invasion (LVSI), lymph node metastasis, and tumor size were significantly associated with DFS. Multivariate analysis further demonstrated that the SII-PNI score, lymph node metastasis, and FIGO stage remained independently associated with longer DFS in patients with early-stage cervical squamous cell carcinoma (ESCC).

**Table 3:** Univariate and multivariate Cox regression analysis for risk of recurrence.

Clinicopathological features	Univariate analysis	*P*	Multivariate analysis	*P*
	HR (95%CI)		HR (95%CI)	
Age (y)		0.593		–
<45	Reference		–	–
≥45	1.11 (0.76 ~ 1.62)		–	–
BMI		0.925		
< 24	Reference		–	–
≥ 24	1.05 (0.64 ~ 1.74)		–	–
FIGO 2018 n(%)		<0.001		<0.001
IA	Reference			
IB	3.21 (1.64 ~ 6.29)		2.80 (1.40 ~ 5.58)	
IIA1	4.42 (2.32 ~ 8.43)		3.37 (1.72 ~ 6.60)	
SII-PNI		0.004		
0	Reference		Reference	<0.001
1	2.24 (1.24 ~ 4.04)		1.97 (1.08 ~ 3.58)	
2	5.85 (3.28 ~ 10.43)		4.75 (2.63 ~ 8.58)	
LVSI		<0.001		0.074
No	Reference		Reference	
Yes	2.13 (1.43 ~ 3.19)		1.51 (0.96 ~ 2.38)	
Lymph node metastases		0.006		0.003
No	Reference		Reference	
Yes	2.58 (1.74 ~ 3.83)		2.19 (1.37 ~ 3.49)	
Tumor size (cm)		<0.001		<0.001
≤ 2	Reference		Reference	
> 2	3.24 (2.19 ~ 4.80)		2.58 (1.74 ~ 3.83)	
Chemotherapy		0.505		–
No	Reference		–	–
Yes	0.83 (0.48 ~ 1.43)		–	–
Radiotherapy		0.88		–
No	Reference		–	–
Yes	0.97 (0.63 ~ 1.49)		–	–

BMI, Body mass index; SII, systemic immune- inflammation index; PNI, prognostic nutritional index; PLT, platelet count; NEU, neutrophil count; LYM, lymphocyte count.

### 3.7 Incremental predictive value of SII-PNI score

To further evaluate whether the SII-PNI score adds prognostic value beyond standard clinicopathological factors, we compared the predictive performance of a Model 1 (including FIGO stage, lymphovascular invasion, and tumor size) with a combined model (Model 1 + SII - PNI score). As shown in **Table 4**, the addition of the SII-PNI score significantly improved the model’s discriminatory ability, with the C-index increasing from 0.710 (95% CI: 0.664 ~ 0.757) to 0.744 (95% CI: 0.698 ~ 0.790) (P = 0.038). Furthermore, the continuous net reclassification improvement (NRI) was 0.393 (95% CI: 0.065 ~ 0.785, P = 0.009), and the AIC value decreased from 1089.3 to 1066.6, indicating that the SII-PNI score significantly improved the risk stratification and model fit for predicting DFS in patients with early-stage cervical cancer.

**Table 4:** Incremental prognostic value of adding SII-PNI to clinicopathological features for predicting recurrence in ESCC.

Model	Variables	AIC	C-index	NRI
Model 1	FIGO stage, lymphovascular invasion, tumor size	1089.3	0.710 (95% CI: 0.664 ~ 0.757)	Reference
Model 2	Model 1 + SII-PNI	1066.6	0.744 (95% CI: 0.698 ~ 0.790)	0.393 (0.065 ~ 0.785)

## 4 Discussion

This study explored the utility of a combined SII - PNI score for evaluating postoperative recurrence risk in patients with ESCC. Elevated SII and decreased PNI were both significantly correlated with an increased likelihood of recurrence. When integrated into a composite SII - PNI score, risk stratification was further improved, demonstrating superior predictive performance compared with either marker alone (AUC: 0.743). Multivariable analysis confirmed that the SII-PNI score remained an independent predictor of recurrence risk. Although the AUC (0.743) indicated moderate discriminative ability, the composite score outperformed individual markers. Crucially, DCA further highlighted its clinical utility, demonstrating a superior net benefit across a wide range of threshold probabilities compared to single markers or default strategies. Collectively, these findings suggest that the SII-PNI score serves as a cost-effective tool complementing traditional pathological factors, thereby optimizing decision-making regarding adjuvant therapy and surveillance intensity without increasing unnecessary interventions.

Accumulating evidence indicates that systemic inflammation ~ related indices are closely linked to adverse clinical outcomes across cervical cancer and various solid tumors, likely reflecting a disequilibrium between enhanced pro−tumor inflammatory responses and compromised anti−tumor immunity. Chen et al. reported that SII functioned as an independent unfavorable prognostic factor in patients with advanced cervical cancer (8). In ESCC, Matteo Bruno et al. reported that increased SII values were significantly correlated with an elevated risk of tumor recurrence (9). K. Holub reported that SII was significantly associated with shorter PFR in patients with cervical cancer (18). On the other hand, tumor initiation and progression are also significantly influenced by the host’s nutritional status (19). Calculated using serum albumin levels and peripheral lymphocyte counts, the prognostic nutritional index serves as a composite indicator reflecting nutritional status and immune function. Accumulating studies have revealed a close association between PNI and survival outcomes across a wide spectrum of malignancies (20–22). For instance, Guo et al. identified PNI as an independent prognostic indicator in cervical cancer (20). In addition, PNI has been shown to predict survival and treatment response in patients with head and neck squamous cell carcinoma (21). In addition, Zhang et al. showed that decreased PNI independently predicted tumor recurrence in patients with thyroid cancer (22). Nevertheless, reliance on single biomarkers may be insufficient for prognostic assessment, as such measures do not fully reflect the complex interactions among systemic inflammation, nutritional status, and immune capacity. Consequently, composite scoring approaches that integrate inflammatory and nutritional parameters, including the SII-PNI score, may provide more comprehensive and complementary risk stratification. An increasing body of evidence has demonstrated the prognostic value of the SII−PNI score in a wide range of malignant tumors (16, 17, 23, 24). However, its value in predicting postoperative recurrence in ESCC has not yet been clearly defined. On this basis, we assessed the prognostic value of the SII-PNI score in a surgically managed ESCC cohort to identify patients at heightened risk of recurrence.

The SII-PNI score, derived from routinely assessed blood parameters (neutrophils, lymphocytes, platelets, and serum albumin), comprehensively reflects systemic inflammation, immune function, and nutritional status. Our finding that an elevated SII-PNI score is independently associated with an increased recurrence risk can be attributed to several biological mechanisms. Specifically, pro-tumorigenic neutrophils (N2-like phenotype) promote angiogenesis, stromal remodeling, and metastasis, while also suppressing anti-tumor immune responses through factors like MMP-9, VEGF, IL-6 (25–29). Lymphopenia, as reflected by decreased lymphocyte counts, signifies impaired cellular immunity and surveillance, impacting treatment tolerance and increasing infection risk (30, 31). Platelets facilitate immune evasion and promote metastasis by interacting with circulating tumor cells and activating cancer stemness pathways (32, 33). Lastly, serum albumin, a marker of both nutritional status and inflammation, can be suppressed by chronic inflammation, leading to hypoalbuminemia, which compromises immune surveillance and treatment tolerance, thereby increasing recurrence risk (34–37).

This study possesses several strengths, including its focus on a surgically treated early-stage cohort and the use of objective, readily accessible biomarkers. Nevertheless, several limitations warrant acknowledgment. First, despite the inclusion of multicenter data to improve generalizability, the retrospective design inherently entails a risk of selection bias. Second, residual confounding from unmeasured variables, such as occult infections, concomitant medication use, or underlying inflammatory conditions may have affected laboratory parameters and thereby introduced potential bias. Third, the optimal cutoff values for the SII and PNI were determined within this specific cohort. Although we performed internal validation using bootstrapping (1,000 resamples) which demonstrated the robustness of our findings (minimal optimism bias), external validation in independent, large-scale populations is still essential before these thresholds can be broadly applied in routine clinical practice. Fourth, in this study, the analysis was confined to patients with squamous cell carcinoma, and therefore the applicability of our findings to other histological subtypes, including adenocarcinoma and adenosquamous carcinoma, should be interpreted with caution. Fifth, while the combined SII-PNI score demonstrated improved predictive value compared to single indices, the calculated reflects a moderate discriminatory power. Consequently, this score is best utilized as an adjunct to, rather than a replacement for, established clinical and pathological prognosticators. In addition, biomarker assessments. Sixth, our analysis was limited by the non-inclusion of other crucial variables known to affect recurrence risk in early-stage cervical cancer, including surgical margin status and parametrial invasion. Future research necessitates a more comprehensive collection of such vital clinical and pathological information to enhance prognostic models. In addition, the median follow-up time was 31.6 months. Although this duration covers the peak period for recurrence in early-stage cervical cancer, it may be insufficient to fully evaluate late recurrence patterns. he median follow-up time was 31.6 months. Although this duration covers the peak period for recurrence in early-stage cervical cancer, it may be insufficient to fully evaluate late recurrence patterns. Therefore, further studies with longer follow-up periods are warranted to validate the long-term prognostic value of the SII-PNI score.

Finally, biomarker assessments were conducted at only one time point, potentially limiting the ability to reflect dynamic changes in systemic inflammatory status or nutritional condition over time. Further investigations incorporating repeated or longitudinal assessments may help improve the prognostic accuracy of these indices.

## 5 Conclusion

This study demonstrates that the joint use of SII and PNI enables a broader assessment of inflammatory burden and nutritional ~ immune condition in individuals with ESCC. Patients presenting with higher composite SII−PNI scores showed a markedly greater propensity for postoperative recurrence. Prospective investigations with larger cohorts across multiple centers are needed to further substantiate the stability and broader applicability of this composite biomarker approach prior to clinical implementation.

## Data Availability

The data analyzed in this study is subject to the following licenses/restrictions: Available upon reasonable request from the corresponding author. Requests to access these datasets should be directed to burabyam@163.com.
