# Supplementary material for: Predictive value of the systemic immune-inflammation index combined with the prognostic nutritional index for postoperative recurrence in early-stage cervical cancer: evidence from a multicenter cohort study
Source: Front Oncol. 2026 Mar 5;16:1792373. doi: 10.3389/fonc.2026.1792373 (PMC12999807; doi:10.3389/fonc.2026.1792373)
Supplement: Supplementary file 1 [file DataSheet1.docx]

Supplementary Materials

Table S1. Relationship of SII, PNI, and their Combined Index with Recurrence in Multivariate Cox Regression Models.

| Variables | Model1 | | Model2 | |
| --- | --- | --- | --- | --- |
|  | HR (95%CI) | *P* | HR (95%CI) | *P* |
| SIIS | 1.27 (1.12 ~ 1.43) | <0.001 | 1.17 (1.02 ~ 1.33) | 0.026 |
| PNIS | 0.68 (0.57 ~ 0.81) | <0.001 | 0.78 (0.64 ~ 0.96) | 0.016 |
| SII-PNI |  |  |  |  |
| 0 | Reference |  | Reference |  |
| 1 | 1.62 (0.95 ~ 2.77) | 0.055 | 1.53 (0.89 ~ 2.64) | 0.055 |
| 2 | 3.65 (2.15 ~ 6.20) | <0.001 | 2.97 (1.71 ~ 5.15) | <0.001 |

Mode 1: Unadjust

Mode 2: Adjust Age, BMI, FIGO , radiotherapy, chemoradiotherapy, tumor size, matrix infiltration depth, lymph node metastases, relapseor or distant metastasis.

Figure S1. Dose-response association between SII or PNI indices and risk of recurrence.
